# A k-mer-based bulked segregant analysis approach to map seed traits in unphased heterozygous potato genomes

**DOI:** 10.1093/g3journal/jkae035

**Published:** 2024-02-15

**Authors:** Pajaree Sonsungsan, Mwaura Livingstone Nganga, Meric C Lieberman, Kirk R Amundson, Victoria Stewart, Kitiporn Plaimas, Luca Comai, Isabelle M Henry

**Affiliations:** Program in Bioinformatics and Computational Biology, Graduate School, Chulalongkorn University, Bangkok 10330, Thailand; Department of Plant Biology and Genome Center, University of California, Davis, Davis, CA 95616, USA; Department of Plant Biology and Genome Center, University of California, Davis, Davis, CA 95616, USA; Department of Plant Biology and Genome Center, University of California, Davis, Davis, CA 95616, USA; Department of Plant Biology and Genome Center, University of California, Davis, Davis, CA 95616, USA; Omics Science and Bioinformatics Center, Faculty of Science, Chulalongkorn University, Bangkok 10330, Thailand; Advanced Virtual and Intelligent Computing (AVIC) Center, Department of Mathematics and Computer Science, Faculty of Science, Chulalongkorn University, Bangkok 10330, Thailand; Department of Plant Biology and Genome Center, University of California, Davis, Davis, CA 95616, USA; Department of Plant Biology and Genome Center, University of California, Davis, Davis, CA 95616, USA

**Keywords:** potato breeding, seed development, bulked segregant analysis, k-mer, trait mapping, Plant genetics and genomics

## Abstract

High-throughput sequencing-based methods for bulked segregant analysis (BSA) allow for the rapid identification of genetic markers associated with traits of interest. BSA studies have successfully identified qualitative (binary) and quantitative trait loci (QTLs) using QTL mapping. However, most require population structures that fit the models available and a reference genome. Instead, high-throughput short-read sequencing can be combined with BSA of k-mers (BSA-k-mer) to map traits that appear refractory to standard approaches. This method can be applied to any organism and is particularly useful for species with genomes diverged from the closest sequenced genome. It is also instrumental when dealing with highly heterozygous and potentially polyploid genomes without phased haplotype assemblies and for which a single haplotype can control a trait. Finally, it is flexible in terms of population structure. Here, we apply the BSA-k-mer method for the rapid identification of candidate regions related to seed spot and seed size in diploid potato. Using a mixture of F_1_ and F_2_ individuals from a cross between 2 highly heterozygous parents, candidate sequences were identified for each trait using the BSA-k-mer approach. Using parental reads, we were able to determine the parental origin of the loci. Finally, we mapped the identified k-mers to a closely related potato genome to validate the method and determine the genomic loci underlying these sequences. The location identified for the seed spot matches with previously identified loci associated with pigmentation in potato. The loci associated with seed size are novel. Both loci are relevant in future breeding toward true seeds in potato.

## Introduction

Cultivated and natural populations of potato (*Solanum tuberosum*) display great phenotypic and genomic diversity (Hardigan *et al.* 2017), only part of which is captured by highly heterozygous commercial clones. Breeding is complicated by the autotetraploid genome of modern cultivated varieties. There is great interest in constructing inbred diploid parental varieties and using these to produce F_1_ hybrids that can be distributed as botanical seed (Almekinders *et al.* 2009; Jansky *et al.* 2016). Central to this strategy is the use of genetic haploid inducers. These are diploids of the subspecies *phureja* that result in maternal-only progeny when crossed to wild-type (WT) clones.

Haploid inducers produce haploids when crossed to diploid WT clones or dihaploids when used to pollinate tetraploid cultivars (Hougas *et al.* 1958; Hermsen and Verdenius 1973). Modern haploid inducers share a common ancestry (Gabert 1963; Hermsen and Verdenius 1973; Hutten *et al.* 1990). They express a dominant anthocyanin marker, the embryo spot, which facilitates the early identification of dihaploids and true hybrids, by visual observation of the seed. The presence of the embryo seed spot is a critical component in the identification of haploids following crossing to haploid inducers from the Phureja group, without which screening for the presence of dihaploids would be significantly more challenging.

Embryo spots result from the deposition of anthocyanins in the cotyledonary axils (De Jong 1991). Color in potatoes is a moderately complex trait determined by several loci (Zhang *et al.* 2017; Riveros-Loaiza *et al.* 2022), spread over most chromosomes (Riveros-Loaiza *et al.* 2022). Different allelic combinations result in various color phenotypes (Jung *et al.* 2009; Zhang, Jung, *et al.* 2009, Zhang *et al*. 2020; Bonar *et al.* 2018). Additionally, other loci govern color patterning. For example, color can be restricted to tuber eyes, tuber flesh, tuber skin, petals, floral abscission zone, nodes, stems, and abaxial or adaxial leaf surfaces (De Jong 1991; Ortiz and Golmirzaie 2003; Jung *et al.* 2009; Zhang, Jung, *et al.* 2009). It can also be expressed in the embryo, which results in a spot visible in the seed. Specifically, the presence of the embryo spot is determined by the combination of 3 loci called P (purple) and R (red), as well as B. The P locus encodes a flavonoid 3′,5′-hydroxylase (chromosome 11) and is necessary for the production of purple pigments (Jung *et al.* 2005). The R locus (which used to be named D) encodes dihydroflavonol 4-reductase (chromosome 2) and is necessary for the production of red pigments (Zhang, Cheng, *et al.* 2009). P is epistatic to R (Hermsen and Verdenius 1973). Finally, when P and R are present and pigments are produced (Dodds and Long 1955; Endelman and Jansky 2016), the B locus controls the patterning of pigmentation and specifically deposition to the seed spot (Dodds and Long 1955). The B locus is closely linked to locus F (Dodds and Long 1955), which was more recently mapped to chromosome 10 (van Eck *et al.* 1994). Alleles *B^c^* or *B^d^* are necessary for the presence of the embryonic seed spot. Haploid inducer IvP35 is homozygous for the embryo spot genes (Hermsen and Verdenius 1973).

Another polymorphism of interest is seed size. Reliance on botanical seed requires that crosses between inbred parents produce seed that germinates and emerges readily after sowing. Seed size is directly related to emergence, establishment, and canopy size (Moles and Westoby 2004). Finally, seed size affects how commercial seeds can be handled and planted. The genetics of seed size has been investigated in tomato, leading to the identification of several quantitative trait loci (QTLs) (Orsi and Tanksley 2009; Khan *et al.* 2012), including the Seed Weight 4.1 (or Sw4.1) locus located on chromosome 4, possibly associated with the function of an *ABC transporter gene* (Orsi and Tanksley 2009; Khan *et al.* 2012). In potato, many QTL mapping and genome-wide association studies (GWAS) have been performed in both tetraploid and diploid populations to identify loci connected to many traits (Young 1996; Bradshaw *et al.* 2008; Byrne *et al.* 2020; Prodhomme *et al.* 2020; Yuan *et al.* 2020; Naeem *et al.* 2021; Alvarez-Morezuelas *et al.* 2023). However, there are no reports identifying genes or loci controlling seed size in potato.

To study the inheritance of both traits, we have hybridized diploid IvP35 (a haploid inducer strain) to GND, a diploid, nonhaploid inducer accession of *phureja* with contrasting traits. In the derived F_1_ and F_2_ populations, we phenotyped color and seed size. To map these traits, we subjected 81 progenies to whole-genome sequencing. Both parents displayed considerable heterozygosity complicating the analysis of haplotypes required for efficient mapping. To overcome this problem, we compared k-mers, arbitrary DNA sequences of k length, between bulked sets of progeny with different traits (Michelmore *et al.* 1991; Sims *et al.* 2009; Nordström *et al.* 2013; Akagi *et al.* 2014; Prodhomme *et al.* 2020). In this case, the entire population was divided into 2 bulks since the 2 traits were binary and there were no extreme trait values. The method highlighted genomic regions involved in color and seed size determination that we could not readily identify by conventional mapping. This bulk strategy allows this analysis to be performed on a small population of individuals from different generations.

## Methods

### Plant materials

Potato clones IvP35 (PI 584995), which is homozygous for the embryo spot (purple) marker genes, and GND (PI 258855), which does not carry the purple spot marker, were obtained from the USDA Plant Germplasm center as in vitro plantlets and as seed, respectively. Both IvP35 and GND are diploid (2*n* = 2*x* = 24). Seeds were germinated and propagated aseptically in half-strength MS media, and in vitro plantlets were transplanted in the greenhouse. Their diploidy was verified by flow cytometry. GND was crossed as the female parent to IvP35. Next, 52 of the resulting diploid F_1_ plants were grown in the greenhouse. To reduce chances of biased marker inheritance caused by the presence of less than 4 self-incompatibility alleles, pollen from these F_1_ plants was harvested and pooled and used to pollinate emasculated flowers of the same pool of F_1_ plants. At least 1 berry from the intercrossed F_1_ flowers was collected from each of the F_1_ plants and its seeds were collected to obtain the F_2_ lines.

### Seed germination and crosses

Seeds that were at least 3 months old were germinated by soaking them in 1,500-ppm GA3 (gibberellic acid) for 24–48 h at room temperature to break any residual dormancy (Lam and Erickson 1966; Lam 1968). The seeds were then rinsed in water and washed in soapy bleach solution (50% bleach with either 0.5% Tween-20 or Triton X-100) for up to 10 min. The seeds were rinsed in water and plated on half-strength MS media (0.5× MS with vitamins, 0.5% sucrose, and 0.7% phytoagar). Germination was performed under cool and dark conditions where the highest germination rates were obtained (Lam and Erickson 1966; Lam 1968). Pollen was extracted by harvesting anthers just before or right after light browning at the tip. Anthers were placed on a filter paper and left to dry for 24–48 hr. Pollen was then extracted using a vibrating rod (VegieBee) on the folded filter paper, collected in 1.5-ml tubes, and stored at 4°C or used immediately. Only pollen less than 2 weeks old was used for pollination. For pollination, female flowers were emasculated by removing the anthers at the start of yellowing. Pollen was then placed on the stigmas of these flowers either immediately or up to 3 days after emasculation, depending on flower maturity. Fruits were harvested at around 30 days and further ripened on the lab bench for up to 30 more days. Seeds were extracted from ripe fruits, rinsed with tap water, washed in 25% bleach for 10 min, rinsed, and placed on a filter paper to dry. The bleach wash also bleached the seed coat and increased the visibility of the seed embryo spot. Seeds were stored for at least 3 months on the lab bench before being sown.

### Seed phenotyping

Both F_1_ and F_2_ seeds were characterized in terms of size and for the presence of the purple seed spot. Spotted and nonspotted seeds were identified visually simply by recording the presence or absence of the dark embryo spot. Seeds were divided into large (L) and small (S) bulks visually as well. In summary, each seed was assigned to 1 of 4 categories based on this very simple visual assessment: large spotted (LSP), large nonspotted (LNS), small spotted (SSP), and small nonspotted (SNS) after a rapid visual assessment.

### Determination of nuclear genome content

Ploidy was determined using a modified protocol based on the method of Rayburn *et al* (1989) and Bino *et al* (1993). Young leaves of in vitro plants were chopped in chopping buffer to release nuclei and the tissue debris was filtered into a test tube by passing the chopped leaf suspension liquid through the cell strainer (35-μm mesh size) Snap Cap of a Falcon Round-Bottom Polystyrene Test Tubes (catalog number 352235). A BD Biosciences *FACScan* flow cytometer or Beckman Coulter *Cytoflex* flow cytometer was used for ploidy analysis. Red polenta (Amundson *et al.* 2023) was used as the tetraploid control while GND and IvP35 were used as the diploids controls. At least 5,000–10,000 events were used to call the ploidy.

### Sequence analysis

IvP35 was previously sequenced to around 30× coverage (Amundson *et al.* 2021). Leaf tissue from each of the F_1_ and F_2_ lines and GND were used for separate DNA extractions, library preparation, and sequencing using Novogene Corporation's internal protocols. Paired-ended 150-bp reads were obtained on the Illumina NovaSeq 6000 platform for an average of 10× read coverage per sample. Custom Python scripts were used to demultiplex the reads and remove the adapter sequences (https://github.com/Comai-Lab/allprep). Independent libraries were obtained for each F_1_ and F_2_ individual. The list of samples sequenced can be found in (Supplementary Table 1).

### Trait mapping

Reads from high-throughput sequencing were mapped to the potato reference genome assembly version DM v6.1 (Pham *et al.* 2020) using the BWA mem algorithm (Li 2013) and default parameters. Variable positions were called using BCFtools version 1.10.2 (Danecek *et al.* 2021). Only reads with mapping quality greater than 30 were used, and all duplicate variants were removed (BCFtools norm -d all). Aligned reads from GND and IvP35 were compared to find genomic variants between the 2 parents. Sites with genotype inference score qualities lower than 100 or with read depth higher than 70 (2.3× genomic average) or less than 25 (0.83× genomic average) were excluded. Positions that were homozygous for different alleles in IvP35 and GND were retained as parental SNPs (618,599 positions).

To genotype the F_2_ lines in terms of parental contributions, the genome was first partitioned into 100-kb consecutive nonoverlapping bins. For each individual genotype was based on pooled information from all SNPs located within each bin. The minimum number of informative reads covering SNPs in a bin was set at 50. The genotype of bins for which fewer than 50 informative reads were available were left unassigned (NA). For bins with sufficient informative reads, a locus was called homozygous when more than 95% of reads were assigned to 1 parent and heterozygous when more than 5% but less than 95% of the informative reads were assigned to both parental genotypes. A total of 4,039 with sufficient data remained and were used as markers for mapping using the QTL (R/qtl) package version 1.41-6 (Broman *et al.* 2003; Arends *et al.* 2010). A map of the position of these bins is presented in Supplementary Fig. 1. Standard data cleanup outlined in the R/qtl manual was followed (Broman and Sen). Recombination frequencies were calculated and presented in Supplementary Fig. 2. Mapping was performed using the binary model for single QTL mapping (scanone function). The logarithm of the odds threshold for significance was set by running permutation tests (*N* = 1,000), as recommended in the R/qtl manual (Broman and Sen).

### K-mer counting

Jellyfish, a multithreaded hash-based tool, was used to generate 31-bp k-mers present in our sequencing reads (Marçais and Kingsford 2011). This was performed independently for all samples. Next, k-mer counts were generated for each sample. K-mers that appeared only once in a sample were removed as they are likely to originate from sequencing errors or contamination. Next, the samples were divided into 4 phenotypic bulks [spotted seeds (N = 53) vs nonspotted seeds (*N* = 28) and large seeds (*N* = 38) vs small seeds (*N* = 43)], with each sample represented in one of the seed size bulks and one of the seed color bulks. For each bulk, the k-mer lists from all individual samples were combined into a single list, containing k-mer sequences and counts for that bulk. Bulk k-mer profiles were generated using GenomeScope version 2.0. These profiles were used to identify a minimum k-mer count threshold for each bulk (Ranallo-Benavidez *et al.* 2020). Specifically, k-mers found less than 5 times in a given bulk were removed from the list. Finally, k-mer counts per bulk were merged into a single count table.

### Identification of significantly enriched k-mers

To identify k-mer that exhibited significantly different counts between 2 bulks, we applied a log-likelihood ratio test for nested models (Rahman *et al.* 2018). Briefly, given that each k-mer appears *K_A_* times in bulk A and *K_B_* times in bulk B, and if *N_A_* and *N_B_* are, respectively, the total number of k-mers in bulk A and bulk B, the k-mer counts are assumed to be Poisson distributed with rate *K_A_*/*N_A_* and rate *K_B_*/*N_B_* in bulk A and bulk B, respectively. The null hypothesis was that the rate of occurrence of a k-mer in bulk A and bulk B is not different. We added 1 to all k-mer counts to avoid 0 values. We used *P*-values to evaluate the significance of the chi-square statistics and performed a Bonferroni correction to account for multiple testing (Rahman *et al.* 2018). K-mers associated with adjusted *P*-values < 0.01 were retained as significantly enriched in 1 bulk vs the other. Finally, only k-mers exhibiting absolute log2 fold change > 2.5 between the 2 bulks were retained as differential k-mers. This resulted in a list of differentially enriched k-mers for each of the 4 bulks (Table 1).

**Table 1.** Number of significant k-mers in each bulk and how many were specific to each parent.

Category	Seed spot		Seed size	
Bulks	Spot	Nonspot	Large	Small
Number of sequenced individuals	53	28	38	43
# significantly enriched k-mers	8,430	557,021	231,677	6,373
GND-specific k-mers	9	173,056	74,707	3,766
IvP35-specific k-mers	6,679	1,460	10,144	125

### Parental origin of the enriched k-mers

To determine the parental origin of each k-mer identified as significantly enriched in one of the 2 progeny bulks, k-mers were obtained from the parental reads as described above. Next, for each of the enriched k-mers, we compared k-mer abundance in the 2 parental readsets. A significantly enriched k-mer was classified as parent specific if it was present in 1 parent but completely absent in the other. Reads containing these k-mers were mapped to the reference for localization, as described below.

### Genomic location of the enriched k-mers

All sequencing reads that contained at least one of the significant k-mers were identified. To visualize the location of the k-mers significant to each bulk and their relative abundance, we performed dosage analysis as follows. Reads containing the significantly enriched k-mers were mapped to the reference genome of the doubled monoploid potato of S. *tuberosum* Group Phureja DM v6.1 (Pham *et al.* 2020) using BWA version 0.7.17 (Li 2013). Next, we counted the number of reads mapping to each consecutive, nonoverlapping 200-kb bin along the genome, for each bulk. Reads with mapping quality < 40 were discarded. This was performed using the custom Python script bin-by-sam.py (https://github.com/Comai-Lab/bin-by-sam). To reduce bias due to the difference in sample size in each bulk, we normalized the number of reads from the significant k-mers for each bulk to the total number of reads found in the corresponding bulk for each bin (Supplementary Tables 2 and 3).

### Pseudo-phasing of GND SNPs under the chromosome 10 QTL

To characterize the sequence variation present between GND and IvP35 under the seed spot QTL identified on chromosome 10, we first obtained the list of positions that are polymorphic between GND and IvP35, and located on chromosome 10, from 44 Mb to the end of the chromosome. These positions were either homozygous in both parents or heterozygous in GND and homozygous in IvP35. The following filters were applied: IvP35 coverage ranged between 18 and 64, percentage of IvP35 alt allele ≤0.05 OR ≥0.95, GND coverage ranged between 10 and 50, GND alt allele ≤0.05 OR ≥0.95 OR between 0.4 and 0.6, GND and IvP35 not homozygous for the same allele, and variant Phred-scale quality score (QUAL) = 999. Finally, we restricted our search to positions that matched one of the 4 bases in both parents and both alleles (no insertions or deletions). This generated a total of 63,447 positions, located on chromosome 10, from 44 Mb to the end. To calculate the percentage of GND allele in the 2 bulks (spotted and nonspotted), we next pooled all of the reads from the 2 bulks and mapped them to DM v6.1 as described above. We produced an mpileup file using SAMtools and a parsed-pileup file using a custom Python script (https://github.com/Comai-Lab/mpileup-tools), which calculated the percentage of GND allele in each of the 2 bulks, based on the parental genotypes and the percentage of reads that mapped the GND-specific allele (Supplementary Tables 4 and 5).

## Results

### The F_2_ population varies in seed size and color

Clone GND (female), which does not exhibit a seed spot, was pollinated with IvP35 (male), which carries a homozygous, dominant seed purple spot (Hermsen and Verdenius 1973), to generate progeny and observe seed traits. IvP35 exhibits deep purple coloration in flowers, flower/fruit abscission zone, tuber flesh, and skin, stem (nodes and internodes). GND, on the other hand, produces light purple flowers, white tuber flesh, white tuber skin, purple-red abaxial leaf surfaces, and lightly colored green stems.

Surprisingly, only 57% of the F_1_ seeds were spotted. Flow cytometry was used to verify the genome content of the F_1_ lines in the nonspotted lines and confirmed that they were diploid, despite the lack of spot. This is unexpected because IvP35 was bred by Hermsen and Verdenius to be homozygous for the dominant embryo spot factor (Hermsen and Verdenius 1973; Singsit and Hanneman 1987). This suggests that genetic factors in GND might influence the expression of the embryo spot trait.

**Fig. 1.**
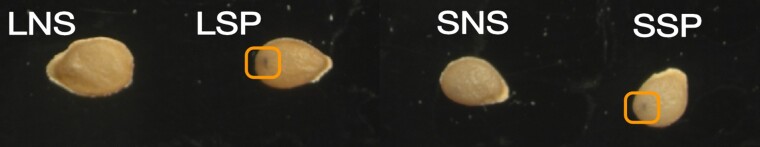
Seed traits. F_1_ and F_2_ seeds were visually separated into 4 categories as follows: SSP, SNS, LNS, and LSP. The spots are highlighted by the boxes.

Flowers from 52 F_1_ plants were intercrossed to produce seed, which we refer to as F_2_ from now on, for simplicity. The F_2_ seed fell into 2 size categories based on visual assessment (Fig. 1). The F_2_ seed also fell into 2 classes based on the presence or absence of the embryo spot. Of 1128 F_2_ seeds observed, 38.4% were spotted and 31% were small. All 4 phenotypic categories, LSP, SSP, LNS, and SNS, were represented. There was no difference in seed size between spotted and nonspotted seeds. To investigate the genetic factors controlling seed size and seed color in this population, 81 samples (8 F1 and 73 F2) were selected representing both large (*N* = 38) and small (*N* = 43), as well as spotted (*N* = 53) and nonspotted (*N* = 28) seed (Table 1 and Supplementary Table 1). Specifically, these 81 samples included 24 LSP seeds, 29 SSP seeds, 14 SNS seeds, and 14 LNS seeds. These seeds were planted, and tissue was collected from the germinated seedlings for DNA extraction and sequencing.

### Parental genotypes are not associated with either trait

To identify QTLs for the traits of interest, Illumina sequencing reads from a total of 81 F_1_ and F_2_ plants were aligned to the potato reference genome assembly version DM v6.1 (Pham *et al.* 2020). A total of 618,599 parental SNPs, which are homozygous in both parents but different between GND and IvP35, were used to characterize the F_2_ plants in terms of parental genotypes. SNP data were merged into 100-kb bins spanning the entire genome (see *Methods* for details). The percentage of heterozygous bins for the F_2_ individuals ranged between 32 and 78%, with a mean of 54%, as is expected from an F_2_ population, and confirming that no haploids were included in this F_2_ population.

We applied the single interval mapping method using a model specific to binary traits for mapping (implemented in the R/qtl package). QTL mapping using the parental SNPs did not identify any significant QTLs for either seed spot or seed size (Supplementary Fig. 3). This is possibly due to the fact that a single parental haplotype controls these traits. For example, if GND is heterozygous for the causative allele, a segregating GND allele regulating the expression of the embryo seed spot would not easily be detected using markers that only differentiate between homozygous parental genotypes. Haplotype-phased parental genotypes would be required to genotype the population at the haplotype level, which is not available for this population and is often not easily obtainable in breeding programs.

### A k-mer-based identification of bulk-specific sequences without a reference sequence

**Fig. 2.**
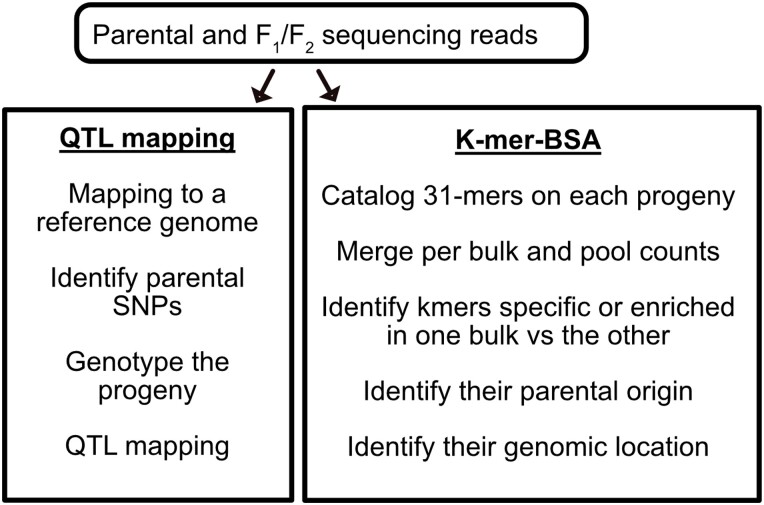
Overview of the 2 analysis pipelines. GND was pollinated with IvP35 to generate F_1_ plants, which were intercrossed to generate an F_2_ population. The plants were assigned to color and size categories based on visual observation of each seed. The F_2_ plants and a few F_1_ plants were sequenced individually using Illumina short-read sequencing. Trait mapping was performed using 2 approaches: QTL mapping based on each plant's parental genotype, and BSA of k-mer counts. For the QTL analysis (left), Illumina short reads were mapped to the potato reference genome assembly, and each individual was genotyped based on parental polymorphisms before QTL mapping. For the BSA-k-mer approach, Illumina short reads were used to generate 31-mer. These k-mers were cataloged based on the abundance in the 2 bulks for each trait. Significantly enriched k-mers were identified and traced back to the original sequencing reads, which were mapped to the reference sequence to identify regions of interest.

To overcome this problem, we applied a k-mer-based method instead. For each trait, we split the population into 2 bulks and compared 31-mer counts between paired bulks to identify k-mers associated with the 2 specific traits (Fig. 2). The k-mer approach may reduce biases in variant calling and, at least initially, does not require a reference genome. This method is thus more flexible and can in theory distinguish all 4 parental haplotypes.

**Fig. 3.**
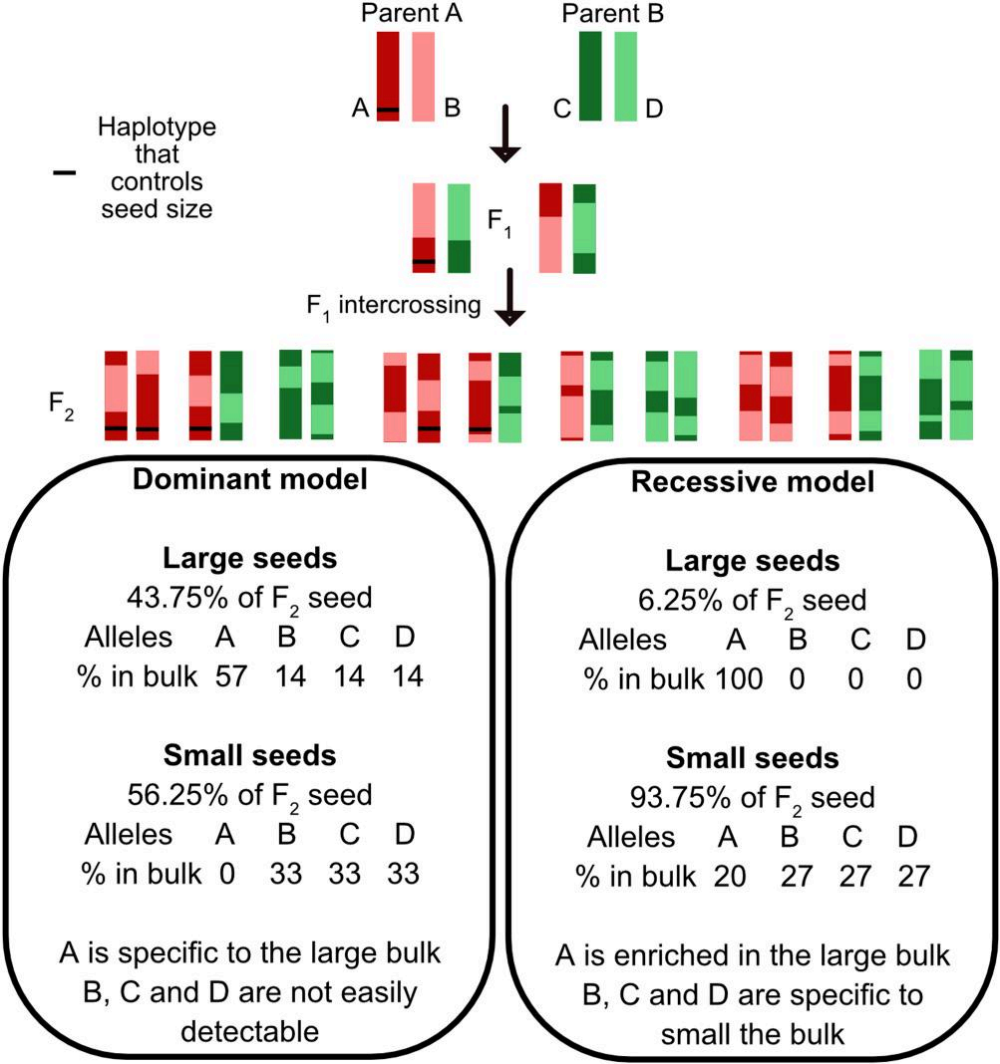
Expected k-mer enrichment patterns based on the genetic mode of action of the causal loci. For any given locus controlling the trait, k-mer enrichment in one bulk or the other will vary depending on whether the allele acts recessively or in a dominant fashion. L represents the large seed genotype, and S represents the small seed genotype.

Figure 3 illustrates the principles underlying the identification of bulk-specific k-mers and highlights expectations, depending on the mode of action of loci responsible for these traits. For example, if we assume that large seed size is controlled by a single haplotype in one of the 2 parents (allele A), we can envision 2 simple situations. If the action of this allele is dominant, any F_1_ or F_2_ seed with at least 1 copy of allele A will be large, while all others will be small. In terms of k-mers, we would expect that k-mers associated with the A alleles will be specific to the large bulk and be readily identified by our pipeline. On the other hand, k-mers associated with the other 3 alleles (B, C, and D) will be more abundant in the small bulk but present in both. Specifically, we expect those k-mers to be approximately 2.4× more abundant in the small bulk (14% vs 33%) and are less likely to be identified in our analysis since we are only retaining k-mers that exhibit a >2.5-fold enrichment in 1 bulk vs the other (see below). If the action of the A allele is recessive, only AA seed will be large. As a result, k-mers associated with the B, C, and D alleles will be specific to the small bulk. K-mers associated with the A allele will be present in both bulks, but they will be significantly enriched in the large bulk compared to the small (100% vs 20% of alleles). They should therefore be identifiable in our analysis as well since we are retaining all k-mers with a 2.5 or higher enrichment in 1 bulk vs the other. In other words, loci underlying a dominant allele are expected to be detected in only one of the 2 bulks while loci underlying a recessive allele are expected to be detectable in both bulks.

Overall, between 2.5 and 4.15% of the initial k-mer sets were significantly enriched in 1 bulk vs the other (see *Methods* for details). Specifically, for the seed spot, we identified 565,451 k-mers that were significantly enriched in 1 bulk vs the other. Of those, 557,021 were enriched in the nonspotted bulk and 8,430 k-mers were enriched in the spotted bulk. Finally, of 557,021 k-mers that were enriched in the nonspotted bulk, 173,056 were GND specific and 1,460 were specific to IvP35. Of the 8,430 k-mers specific to the spotted bulk, 9 were specific to GND and 6,679 were specific to IvP35.

For seed size, 231,677 k-mers were significantly enriched in the large bulk and 6,373 were enriched in the small bulk. Of the 231,677 SNPs enriched in the large-seeded bulk, 74,707 were specific to GND and 10,144 were specific to IvP35. Of the 6,373 SNPs specific to the small-seeded bulk, 3,766 were GND specific and 125 IvP35 specific.

Based on the rationale presented above, these numbers suggest that both the nonspotted and large seed traits could each be controlled by dominant alleles at a single locus that is specific to and heterozygous in the GND parent.

### Identification of genomic regions associated with the enriched k-mers

For species for which a reference genome is available, a last step can be included that enables the genomic localization of the enriched k-mers. In our case, this step also serves as a way to validate the results obtained using the reference-free approach.

**Fig. 4.**
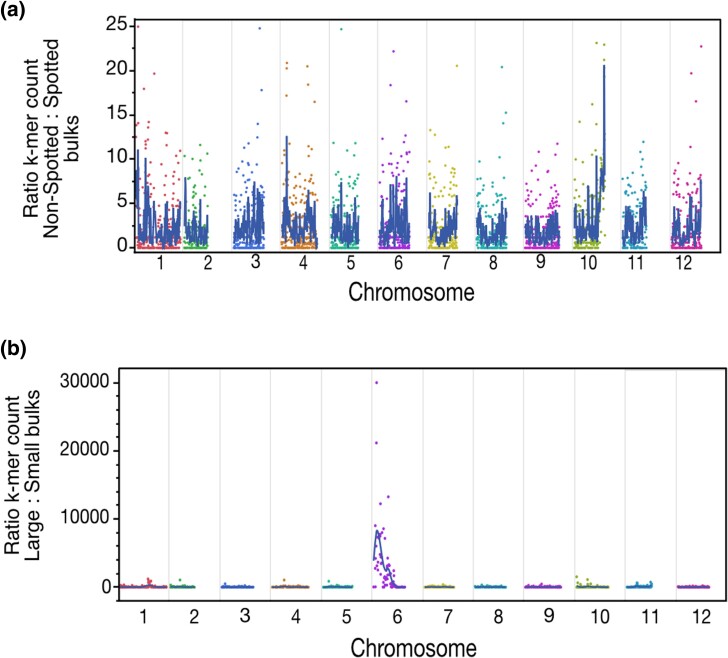
Genomic location of the enriched k-mers. a) Ratio of normalized read counts containing significantly enriched k-mers in the nonspotted vs spotted bulks. One major peak is present on chromosome 10. The signal predominantly originates from enriched k-mers originating from the GND parent in the nonspotted bulk (Supplementary Fig. 4 and Table 1). Taken together, the data presented and the expectations described in Fig. 3 suggest the existence of a GND-specific allele located on chromosome 10, which acts dominantly and sporophytically to produce nonspotted seed. b) Ratio of normalized read counts containing significantly enriched k-mers in the large vs small bulks. One major peak is present on chromosome 6. The signal predominantly originates from enriched k-mers originating from the GND parent in the large bulk (Supplementary Fig. 5 and Table 1). Taken together, the data presented and the expectations described in Fig. 3 suggest the existence of a GND-specific allele located on chromosome 6, which acts dominantly and sporophytically to produce large seed.

To characterize the location of the bulk-specific k-mers, we first identified which of the genomic reads contained the enriched k-mers. We next mapped the original reads containing these k-mers to the DM v6.1 potato genomic assembly (Pham *et al.* 2020). Finally, we derived the distribution of reads significantly enriched in the different bulks (Supplementary Figs. 4 and 5) and their ratio in 1 bulk vs the other (Fig. 4).

Mapping the reads that contained k-mers significantly associated with seed spotting resulted in the identification of 1 clear peak of k-mers enriched in the nonspotted bulk, located at the end of chromosome 10 (47.8 Mb to the end of the chromosome; Fig. 4 and Supplementary Fig. 4). The same peak was also visible in the location of the k-mers enriched in the spotted bulk, but it was smaller (Supplementary Fig. 4). Next, we determined whether these bulk-specific k-mers were specific to one parent or the other (Supplementary Fig. 4b, c, e, and f). When looking at the distribution of the parent-specific k-mers only, the same peak appeared in the GND-specific k-mers but was absent in the IvP35-specific k-mers.

We applied the same approach to identify loci associated with seed size. This resulted in the identification of a wide peak on chromosome 6, only present in the k-mers specific or enriched in the large bulk (Fig. 4 and Supplementary Fig. 5). Based on the percentage of large seeds observed in the F_2_ population (69% large seed), and the presence of a single peak in one bulk vs the other, our results are consistent with a dominant allele that confers increased seed size. Investigation of the parental origin of these bulk-specific k-mers demonstrated that this dominant allele linked to large seed size originated from the GND parent (Supplementary Fig. 5).

### Detailed characterization of the GND haplotype associated with the lack of seed spotting

**Fig. 5.**
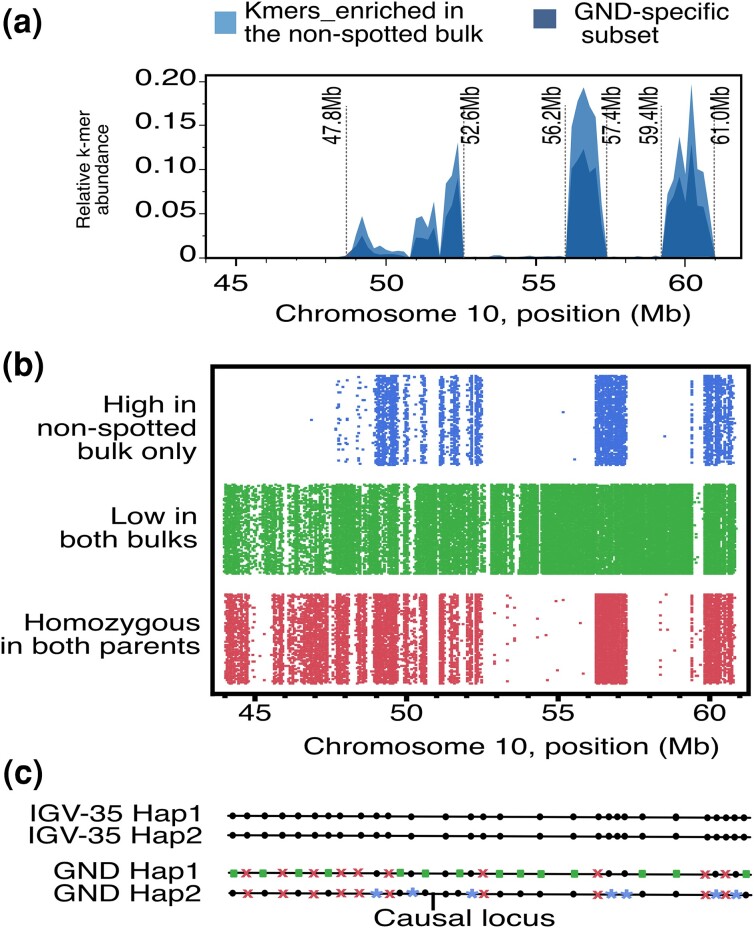
Identification of the GND haplotype harboring the causal gene associated with nonspotted seed. a) Detailed view of the distribution of enriched k-mers at the end of chromosome 10 (45 Mb to the end), showing normalized counts of all k-mer enriched in the nonspotted bulk (top part of the peaks) and the subset of those that are specific to the GND parent (bottom part of the peaks). b) Distribution of parental SNPs in the QTL region. Parental SNPs were divided into 3 categories. Positions that were homozygous in both parents are represented at the bottom. Positions that were homozygous in IvP35 and heterozygous in GND were further divided into 2 potential haplotype groups: positions for which the percentage of GND allele was similar in the 2 bulks (middle) and positions for which the percentage of GND allele was high in the spotted bulk but low in the nonspotted bulk (top; see Supplementary Fig. 7 for details on how these positions were selected). c) Schematic representation of the distribution of the different GND-specific alleles in the 2 GND haplotypes. Circles represent alleles present in IvP35, crosses represent positions that are homozygous in IvP35, squares represent GND haplotype 1 and asterisks represent GND haplotype 2. The locus harboring the causal gene/factor associated with the lack of seed spot is located on haplotype 2 of GND. Compared to haploid 1, this haplotype contains fewer haploid-specific SNPs, and these SNPs are not evenly distributed across the region of interest.

The enriched k-mers associated with seed size were overall evenly distributed in the identified interval (Supplementary Fig. 6). On the other hand, the k-mers associated with the nonspotted bulk exhibited a nonrandom distribution within the identified interval on chromosome 10 (48–60 Mb; Fig. 5a), motivating the question of whether this represented multiple independent QTLs. Alternatively, this dispersed signal may result from varying frequencies of heterozygosity within GND, rather than recombination within this interval, which would suggest a single QTL.

To distinguish between these 2 hypotheses, we characterized this interval further. We first identified all positions that were polymorphic between GND and IvP35. This corresponded to 2 categories of SNPs: those that are homozygous but different in the 2 parents (Supplementary Fig. 7a) and those that are heterozygous in GND and homozygous in IvP35 (Supplementary Fig. 7b). We further divided this second category of SNPs into GND haplotypes, using the GND allele frequency in the 2 bulks as a proxy. Indeed, we expect that the haplotype that is responsible for the nonspotted phenotype is enriched in the nonspotted bulk while the other haplotype is not (Supplementary Tables 4 and 5). The selection of the 2 types of SNPs is illustrated in Supplementary Fig. 7c. Positions that did not clearly fall into either category were labeled as unclear. Finally, we plotted the physical position of each SNP category (Fig. 5b). Both the homozygous parental SNPs and the SNPs that exhibited differential frequencies in the 2 bulks exhibited uneven patterns of SNP distribution while the SNPs belonging to the second haplotype were overall evenly distributed. Furthermore, the SNPs belonging to haplotype 1 exhibited a pattern that fit the enriched k-mers best, while the distribution of the homozygous SNPs was similar but not identical. Specifically, homozygous SNPs are present in the 44- to 48-Mb region while haplotype 1 SNPs and enriched k-mers were absent in that region. Together, these results are consistent with the hypothesis that the identified QTL corresponds to the whole region, as opposed to several distinct peaks. Further, it suggests that the causal factor is associated with the SNPs identified with GND haplotype 1. The percentage of GND-specific alleles in the 2 bulks is consistent with these findings as well.

## Discussion

Mapping traits of interest can be challenging if the reference genome is either not available or too divergent from the parental genomes at hand. Michelmore *et al.* (1991) demonstrated that randomly generated arbitrary PCR markers could be associated with polymorphic progeny bulks. The bulking approach became widely adopted with the advent of high-throughput sequencing technology. Using k-mers, Sims *et al.* (2009) constructed bacterial phylogenies. Nordström *et al.* (2013) identified mutations by bulking WT and mutant progeny. Akagi *et al.* (2014) identified and mapped the sex-determinant mRNA called OGI by comparing male and female cohorts of *Diospyros lotus*. In the succeeding years, multiple GWAS studies employed k-mer analysis with success in systems with variable quality of reference genomes (Kim *et al.* 2020; Voichek and Weigel 2020). Recently, examples of mapping natural variants in an experimental cross using k-mers are emerging, as the approach is extremely flexible and can be applied to many situations (Nordström *et al.* 2013; Prodhomme *et al.* 2020; Fletcher *et al.* 2021), such as the one presented here with individuals from both the F_1_ and F_2_ generation of a cross between 2 heterozygous parents.

Genome-agnostic association of k-mers to traits provides a reliable solution and a versatile approach to the complexity of the pangenome (Vernikos *et al.* 2015): for example, a trait may depend on genes that are present in some individuals and absent in others and in the reference genome. In our case, we did not detect significant signals using standard parent-based mapping approaches (see *Methods*) but instead were able to identify loci associated with both traits when detecting differential representations of k-mer counts between bulks. Additionally, the k-mer approach is not sensitive to linkage or other expectations based on the reference genome used. It is also able to detect association between traits and structural variants. For example, an insertion could be present in one bulk and not the other or absent in the reference genome, making that source of variation undetectable to conventional mapping while the inserted sequences would be identifiable as enriched k-mers. With no prior knowledge of the factor(s) regulating these traits or their potential mode of action, one cannot predict what type of cross will be most likely to enable their identification. The approach presented here has the potential to capture all model types and distinguish between all haplotypes.

The embryo purple spot in seed results from the deposition of anthocyanins and its expression is controlled by a combination of genes that produce various color phenotypes (Dodds and Long 1955; De Jong 1991). The purple anthocyanins on embryo spots are determined by 2 dominant color genes, B and P (Dodds and Long 1955; Endelman and Jansky 2016). Our analysis identified 1 region associated with color, on the distal arm of chromosome 10 (47.8 Mb to the end). Several previous mapping experiments have found the distal end of chromosome 10 to be associated with this phenotype. The region carries several anthocyanin pathway genes including 2 R2R3-MYB transcription factors and multiple anthocyanin biosynthesis genes (van Eck *et al.* 1994; Jung 2005; Jung *et al.* 2009; Endelman and Jansky 2016; Tengkun *et al.* 2019; Parra-Galindo *et al.* 2021). At first sight, there are 2 genes located in the identified region of chromosome 10 (48–60 Mb) that can be identified as potential causal genes. The first gene is StANTHOCYANIN1 (Soltu.DM.10G020850.1, position 52,601,941), a MYB transcription factor, which controls anthocyanin levels (Bao *et al.* 2022). The second gene is StAN2 (Soltu.DM.10G020820.1, position 52,546,308), also a MYB-domain protein, previously shown to control the degree of anthocyanins in tuber (Jung *et al.* 2009; Zhang *et al.* 2020; Riveros-Loaiza *et al.* 2022).

Screening for dihaploids following crossing to haploid inducers based on the presence of the embryo seed spot is critical, and, in typical tetraploid by diploid crosses, this method is usually reliable. However, the results obtained here suggest that other natural alleles can suppress the expression of the IvP35 homozygous color alleles. Specifically, our data are consistent with the presence of a dominant and epistatic GND allele that regulates the action of the IvP35 embryo spot allele. As a result, we did obtain diploid individuals that carried the IvP35 spot allele but did not exhibit the embryo spot. The identification of this GND-specific color-associated locus provides a clear proof of concept of the bulked segregant analysis (BSA)-k-mer method in potato. It also suggests that, in certain crosses, reliance of the seed spot trait to prescreen for potential haploids in the potato haploid induction crosses might be complicated by the maternal genotype.

Reads containing differential k-mers for seed size mapped to the proximal arm of chromosome 6. The region is still fairly large and more individuals would be needed to narrow the search. There are 44 annotated genes in the peak centered at 4.8–6.0 Mb (Supplementary Table 6). Those whose function can be inferred by homology do not belong to pathways known to regulate seed size (Li and Li 2016; Li *et al.* 2019). In our analysis, the differential reads had a distinct pattern: they originated from the GND parent and displayed enrichment predominantly in the large seed bulk. This could be explained if a GND allele enlarges the seed in an additive or dominant manner.

Because our analyses used a limited number of individuals per bulk, it is possible that some of the regions identified are not actually associated with the traits of interest. The identified QTL will first need to be validated either with a larger population of individuals or by functional validation via fine-mapping of the regions and identification and functional validation of the causal genes or factors. Still, our results provide a starting place to design selection markers from the enriched k-mer sets, even without the availability of a reference genome. For example, selecting k-mers with the high levels of enrichment in 1 bulk vs the other (most biased set of k-mers), but with raw abundance levels consistent with single-copy regions, followed by local assembly of the reads containing these k-mers can provide sufficient information to marker design even in the absence of a reference genome.

In summary, by detecting the enrichment of k-mers derived from sequencing reads, we have mapped loci responsible for anthocyanin pigmentation and seed size. The discovery of a potential repressor of pigmentation GND allele on chromosome 10 provides a useful trait to breeders interested in developing true botanical seed in potato.

## Supplementary Material

jkae035_Supplementary_Data

## Data Availability

Sequence data have been deposited in the National Center for Biotechnology Information Sequence Read Archive: BioProject identifier PRJNA984282. Reads from parental accession IvP35 were previously deposited (SRX10043416). Python scripts used in this study can be found at the Comai Lab GitHub repository (https://github.com/Comai-Lab). Supplemental material available at G3 online.
