# Supplementary material for: A k-mer-based bulked segregant analysis approach to map seed traits in unphased heterozygous potato genomes
Source: G3 (Bethesda). 2024 Feb 15;14(4):jkae035. doi: 10.1093/g3journal/jkae035 (PMC10989861; doi:10.1093/g3journal/jkae035)
Supplement: jkae035_Supplementary_Data [file jkae035_supplementary_data.zip › Supplemental_Figures_G3-2023-404804.pdf]

Supplementary Figures

A K-mer based Bulked Segregant Analysis approach to map seed traits in unphased heterozygous potato genomes

Pajaree Sonsungsan[1], Mwaura Livingstone Nganga[2], Meric Lieberman[2], Kirk Amundson[2], Victoria Stewart[2], Kitiporn Plaimas[3,4], Luca Comai[2] and Isabelle Henry[2]

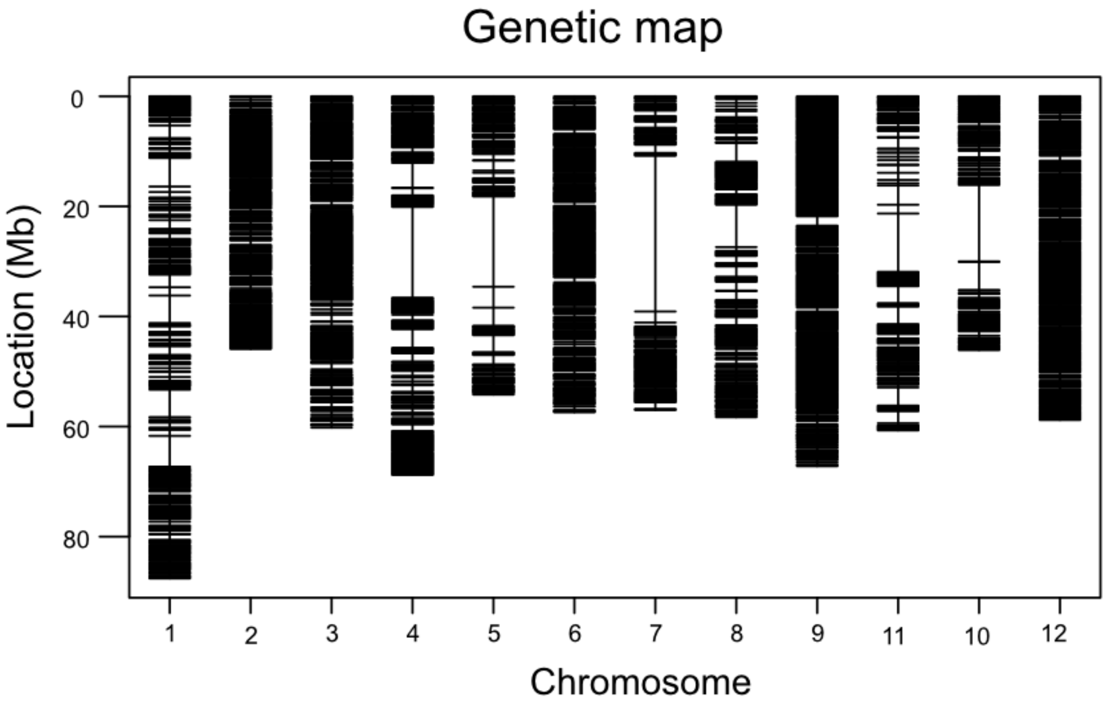

Figure S1. Position of the markers used for QTL mapping using parental genotypes.

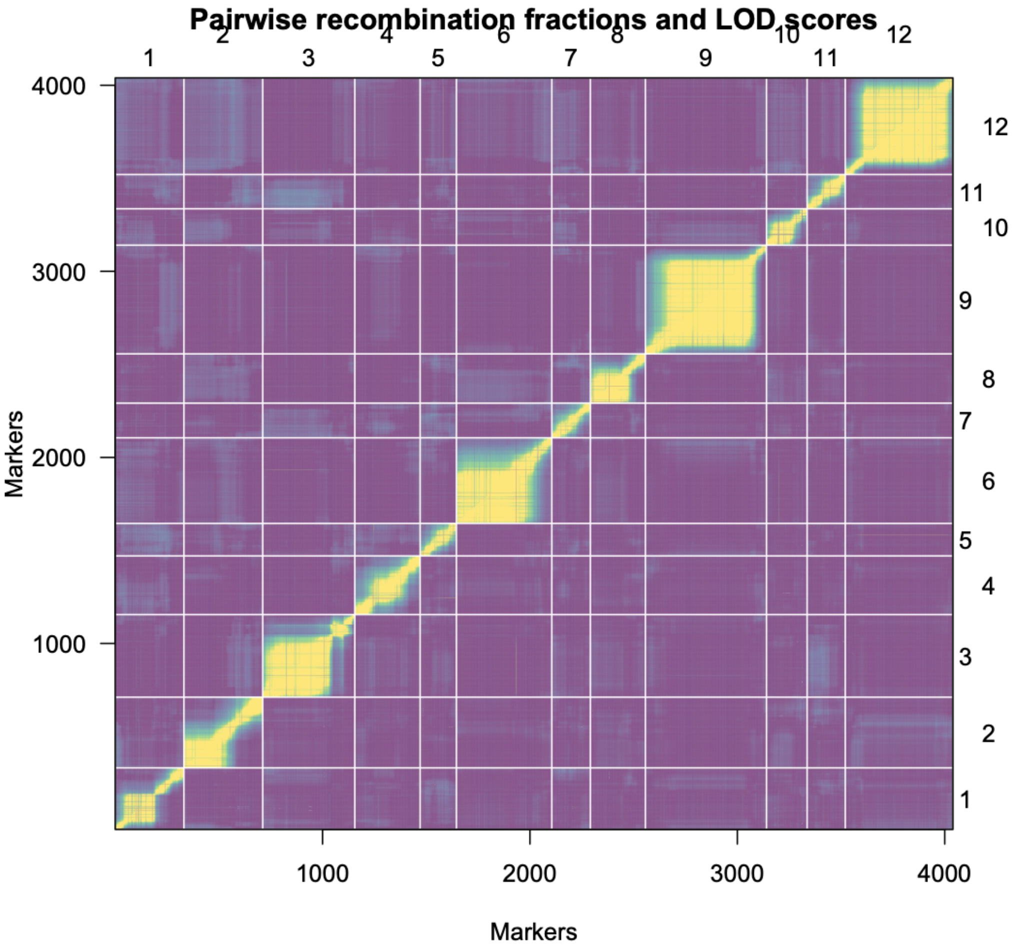

Figure S2. Recombination frequencies

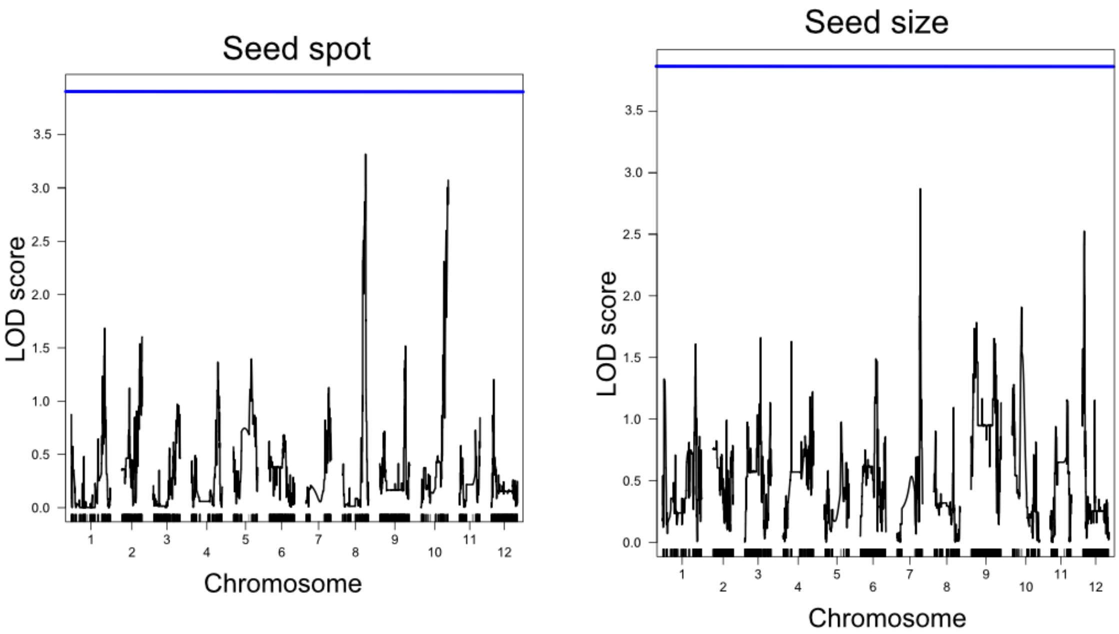

Figure S3. Mapping of the seed size and embryo spot loci using parental genotypes. Genome-wide LOD scores from mapping of the seed spot (left) and seed size (right) binary traits using a binary trait model in R/qtl. The blue horizontal line represents a significance level of 0.05 after a permutation test (N = 1,000).

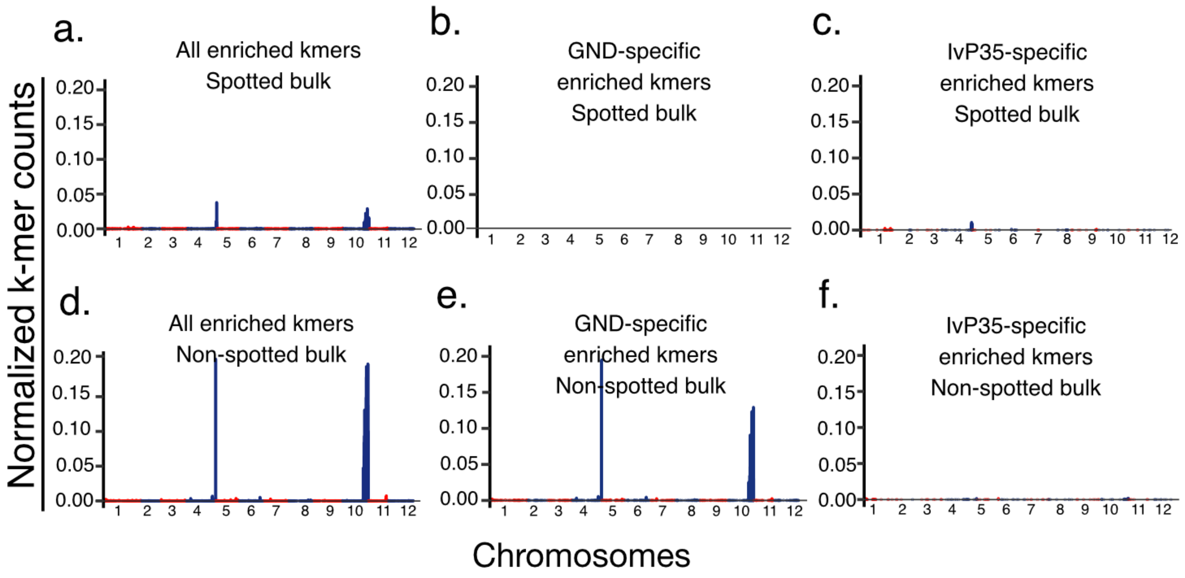

Figure S4. Mapping of the embryo spot loci using k-mers. Distribution of k-mers significantly enriched in one bulk versus the other, depending on the bulk and parental origin. For each plot, the number of reads per 250 kb bin relative to the total number of reads for the corresponding bulk is shown. (a, c, e) significant k-mers in the spotted seed bulk (b, d, f) significant k-mers in the non-spotted seed bulk. (a,b) all reads containing a significantly enriched k-mer (c-f) subset of the reads shown in (a and b) that are also parent-specific.

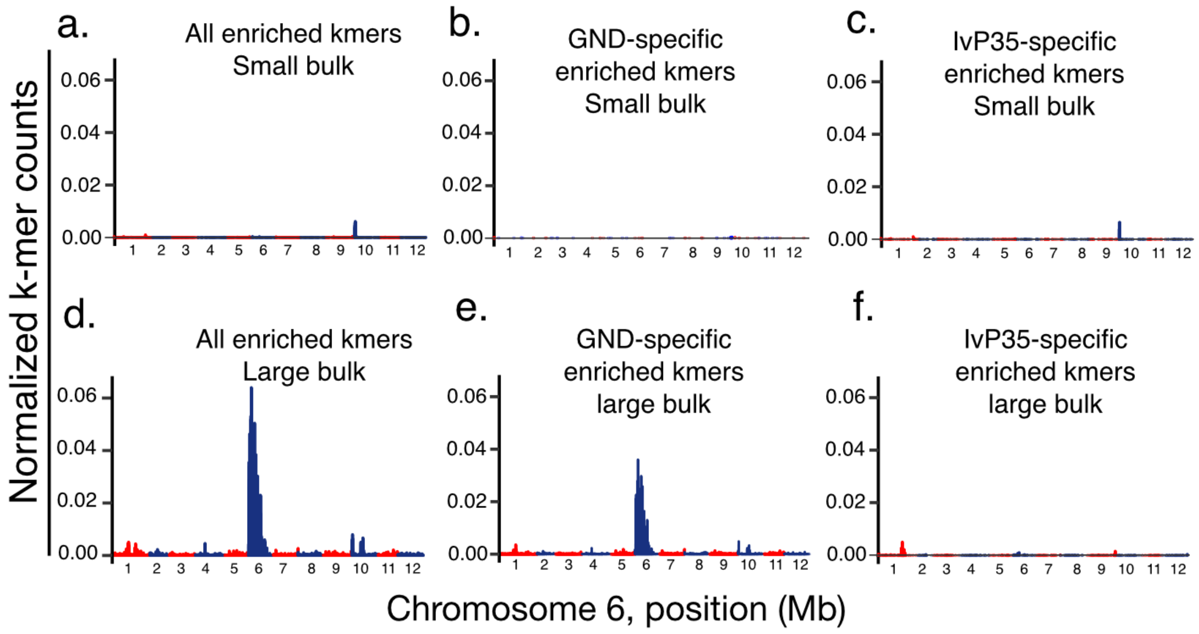

Figure S5. Mapping of seed size loci using k-mers. Distribution of k-mers significantly enriched in one bulk versus the other, depending on the bulk and parental origin. For each plot, the number of reads per 250 kb bin relative to the total number of reads for the corresponding bulk is shown. (a, c, e) significant k-mers in the small seed bulk (b, d, f) significant k-mers in the large seed bulk. (a,b) all reads containing at least one significantly enriched k-mer (c-f) subset of the reads shown in (a and b) that are also parent-specific.

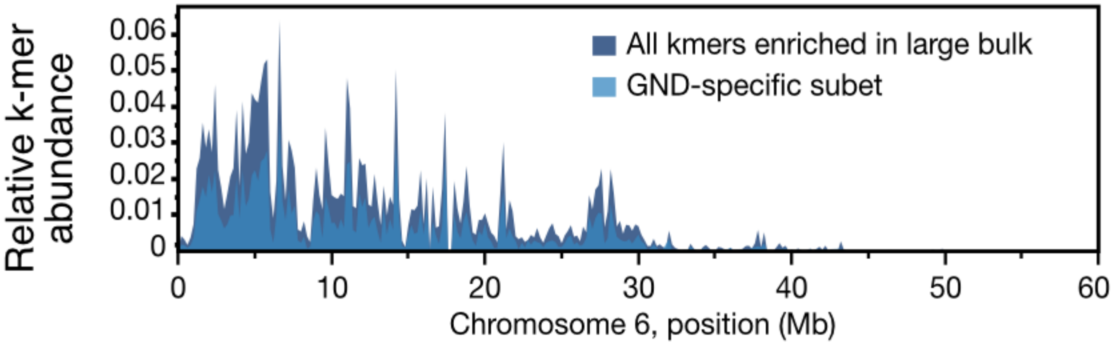

Figure S6. Distribution of enriched k-mers specific to the large bulk. Detailed view of the peaks on chromosome 6, showing normalized counts of all k-mer enriched in the large bulk (dark blue) and the subset of those that are specific to the GND parent (light blue).

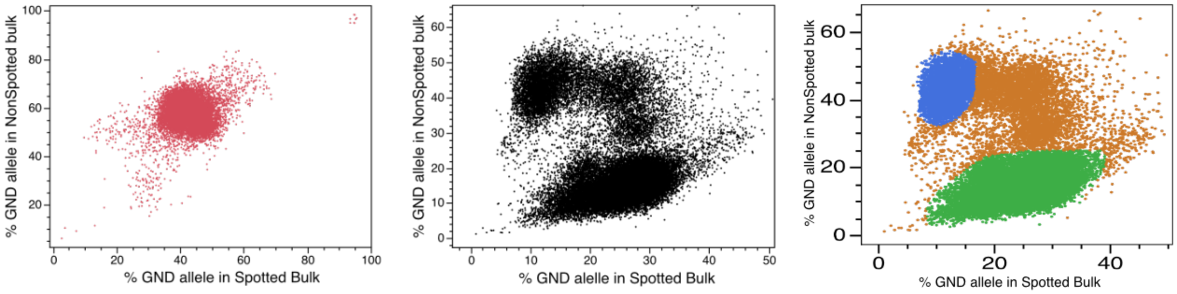

Figure S7. Identification of different types of variant positions within the chromosome 10 QTL associated with seed color. Percentage of GND allele in the spotted and non-spotted bulks at positions that are polymorphic between IvP35 and GND on chromosome 10, 44 Mb to the end. a) Positions that are homozygous in both parents but polymorphic between parents. The mean % GND for those positions was 57.9% for the NS bulk and 42.1% for the spotted bulk. b) Positions that are homozygous in IvP35 and heterozygous in GND c) The percentage of GND allele in the two bulks was used as a proxy to further categorize the SNPs into two haplotypes (blue and green), based on differential selection in the two bulks. SNPS that were intermediate were not selected (orange). The mean % GND for the blue cluster was 43.1% for the NS bulk and 11.8% for the spotted bulk. The mean % GDN for the green cluster was 15.3% for the NS bulk and 26.2% for the spotted bulk (Supplementary Tables S4 and S5).
